# Surface Engineering of Magnetite Nanoparticles with Boronated Polysaccharides Enables Alpha-1-Acid Glycoprotein Binding

**DOI:** 10.1021/acsomega.5c12779

**Published:** 2026-06-17

**Authors:** Kinga Mylkie, Dorota Chełminiak-Dudkiewicz, Anna Ilnicka, Aleksander Smolarkiewicz-Wyczachowski, Marta Ziegler-Borowska

**Affiliations:** † Department of Biomedical Chemistry and Polymer Science, Faculty of Chemistry, 49577 Nicolaus Copernicus University in Torun, Gagarina 7, 87-100 Torun, Poland; ‡ Department of Chemistry of Materials, Adsorption and Catalysis, Faculty of Chemistry, Nicolaus Copernicus University in Torun, Gagarina 7, 87-100 Torun, Poland; § Institute of Advanced Studies, Nicolaus Copernicus University in Toruń, Wileńska 4, 87-100 Torun, Poland

## Abstract

Glycoproteins play essential roles in numerous biological processes, yet their controlled interaction with synthetic materials remains challenging due to structural complexity and heterogeneity of glycan moieties. In this study, starch-based magnetic nanomaterials functionalized with phenylboronic acid were developed and evaluated for pH-dependent interactions with α-1-acid glycoprotein (AGP). Dialdehyde starch (DAS) and carboxymethyl starch (CMS) were functionalized with phenylboronic acid to obtain DAS-PBA and CMS-PBA, which were subsequently used as coatings for magnetite nanoparticles, yielding the corresponding magnetic nanocomposites. The polymers and nanocomposites were comprehensively characterized using spectroscopic, microscopic, and thermal techniques. The functional accessibility of boronic acid groups was assessed using an Alizarin Red S assay. Binding studies demonstrated pH-dependent interactions with AGP, with enhanced binding observed under alkaline conditions, consistent with the behavior of boronic acid–diol interactions. Among the investigated systems, CMS-PBA-based materials exhibited higher AGP binding capacities compared to DAS-PBA-based analogues. Furthermore, partial release of AGP from the magnetic nanocomposites was achieved under mildly acidic conditions, confirming the reversible nature of the interactions.

## Graphical abstract

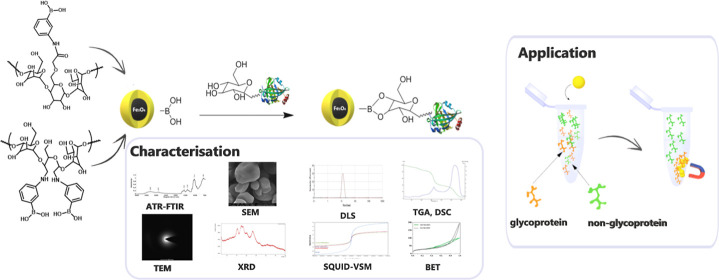

## 1 Introduction

The development of functional nanomaterials for recognizing and enriching glycoproteins is of increasing significance in biomedical diagnostics and proteomics.^1,2^ However, the design of such systems remains challenging due to the low abundance and structural heterogeneity of glycoproteins in complex biological matrices.^3^

Boronic acids are widely recognized for their ability to form covalent bonds with *cis*-diol groups in glycosylated peptides, enabling their selective recognition and capture.^4^ This interaction is pH-dependent; boronate ester formation is favored under alkaline conditions and reversed in acidic media, allowing for controllable binding and release of target biomolecules.^5^ Crucially, these recognition strategies reduce interference from nonglycosylated proteins, enhancing specificity^6–9^ When combined with magnetic nanoparticles (MNPs), boronic acid-based systems gain the advantage of rapid magnetic separation, providing practical and scalable solutions for biomolecular analysis.^10,11^ So far, most boronic acid—functionalized MNPs have relied on dextran or cellulose-based coating to improve colloidal stability and biocompatibility.^12–15^ Apart from these two examples, other natural biopolymers have received little attention as functional matrices for such systems. For example, cellulose-based magnetic nanocomposites functionalized with phenylboronic acid have been investigated for the adsorption of ovalbumin, showing pH-dependent binding behavior with moderate adsorption capacities under alkaline conditions.^16^ Similarly, dextran-coated magnetic nanoparticles bearing aminophenylboronic acid have been reported for antibody separation, demonstrating preferential binding toward glycosylated immunoglobulins compared to nonglycosylated proteins^17^ While these studies confirm the applicability of boronic acid–diol interactions for glycoprotein recognition, they primarily focus on adsorption performance and do not address the functional accessibility of boronic acid groups or the influence of polysaccharide architecture on binding efficiency. Although abundant, renewable, biodegradable, and chemically versatile, starch has not been systematically explored as a coating material for boronic acid-modified nanoparticles.^18,19^ Its structural diversity and ease of chemical modification open opportunities for designing new nanomaterials that are both effective and sustainable. Notably, the high density of hydroxyl groups present in starch may influence the functional accessibility of boronic acid moieties due to potential intramolecular or intermolecular interactions, underscoring the need for systematic evaluation of boronic acid availability in starch-based systems.

This study reports synthesis and characterization of starch-based magnetic nanoparticles functionalized with 3-aminophenylboronic acid (PBA). Two different starch derivatives—dialdehyde starch and carboxymethyl starch—were chemically modified with PBA and used as coatings for magnetite nanoparticles. Their correct formation and structure were confirmed by ATR–FTIR and NMR spectroscopy. The binding performance of the obtained nanomaterials was systematically evaluated, starting with glucose as a model compound and subsequently with alpha-1-acid glycoprotein (AGP)^20,21^ AGP is an acute-phase plasma protein predominantly synthesized in the liver, involved in immunomodulation and in the transport and binding of pharmaceutical agents.^22,23^ Elevated AGP levels are strongly associated with inflammation, infection, and tissue damage, making it an established biomarker in sepsis, autoimmune diseases, and malignancies such as hepatocellular carcinoma^24–26^

The main aim of this study was to design and evaluate a new class of boronated starch-based magnetic nanomaterials for glycoprotein binding. By combining the binding capability of boronic acids with the biocompatibility and chemical versatility of starch, together with the magnetic separability provided by nanoparticle cores, this work explores starch-based platforms as model systems for pH-responsive and reversible interactions with glycoproteins.

## 2 Materials and Methods

### 2.1 Materials

Iron­(III) chloride hexahydrate, ethylenediamine, sodium periodate, epichlorohydrin, (3-(dimethylamino)­propyl)-3-ethylcarbodiimide hydrochloride (EDC), glycerol, diiodomethane, ninhydrin reagent, monochloroacetic acid, α-1-acid glycoprotein, sodium carbonate, sodium acetate, sodium dihydrogen phosphate 12·H_2_O, sodium hydrogen phosphate dihydrate and human serum albumin were purchased from Merck (Darmstadt, Germany). Corn starch purchased from Vitargo (19.25% amylose content, determined using the Amylose/Amylopectin Assay Kit purchased from Megazyme). Iron­(II) chloride tetrahydrate, acetic acid, sodium bicarbonate, sodium hydroxide, sodium carbonate, 3,5-dinitrosalicylic acid, glutaraldehyde, methanol, ethyl alcohol, sodium hydroxide, hydrochloric acid, and acetic acid were purchased from Avantor Performance Materials Poland S.A (POCH, Gliwice, Poland). 3-Aminophenylboronic acid was purchased from Fluorochem. Alizarin Red S was purchased from Warchem. Deionized water was used to prepare the solutions.

### 2.2 Preparation of Dialdehyde Starch (DAS)

The synthesis of dialdehyde starch was conducted in a manner analogous to the procedure described in the literature.^27^ Corn starch (1.5 g) was dissolved in deionized water (30 mL) and thoroughly mixed using a magnetic stirrer (room temperature). To minimize photodecomposition of sodium iodate (NaIO_4_), an aqueous solution (0.70 M, 10 mL) was added to the mixture, and the flask was immediately covered with aluminum foil. The mixture was stirred magnetically at 40 °C for 3 h. Upon completion, the reaction mixture was allowed to cool to room temperature, and the product was precipitated by the addition of acetone (100 mL). The solid was collected by vacuum filtration, washed successively with deionized water (3 × 15 mL) and acetone (50 mL), then air-dried at room temperature for 24 h.

#### 2.2.1 Determination of the Aldehyde Group Content on the Surface of DAS

The content of aldehyde groups was determined using the procedure described in the literature^28^ with slight modifications. Dialdehyde starch (0.1 g) of was mixed with 5 mL of 0.25 M sodium hydroxide (NaOH) solution. The mixture was heated in a water bath at 70 °C until the sample was completely dissolved. After cooling to room temperature, 7.5 mL of 0.25 M hydrochloric acid (HCl), 15 mL of deionized water, and phenolphthalein indicator was added. The solution was then titrated with 0.25 M NaOH. The determination was performed in triplicate for each sample. The aldehyde group content was calculated using the following formula

1 $$ALD\left(\%\right)=\frac{C_{1}V_{1}-C_{2}V_{2}}{m/M}\cdot 100\%$$

where: *C*_1_ and *V*_1_ is the concentration (mol/L) and volume (L) of the solution NaOH; *C*_2_ and *V*_2_ are the concentration (mol/L) and volume (L) of the HCl solution; *m* is the mass of the sample (g), M is the molecular weight of the repeating unit in dialdehyde starch (*M* = 160 g/mol).

### 2.3 Preparation of Dialdehyde Starch Functionalized with 3-Aminophenyl Boronic Acid (DAS-PBA)

3-Aminophenylboronic acid (0.1 g; 0.73 mmol) was dissolved in 3 mL of methanol. Separately, 1.0 g of dialdehyde starch was suspended in 10 mL of methanol and added to the solution. The reaction mixture was stirred at room temperature for 24 h. Then, 3 mL of a methanolic sodium borohydride (NaBH_4_) solution (0.01 M) was added, and stirring was continued for 6 h at room temperature. The resulting solid was separated by filtration, washed with methanol (3 × 5 mL), and dried under vacuum at 30 °C for 24 h.

### 2.4 Preparation of Carboxymethyl Starch (CMS)

Carboxymethyl starch was synthesized by modifying the procedure described in the literature.^29^ Starch (3 g) was suspended in 10 mL of ethanol. A 5 M aqueous sodium hydroxide solution (5 mL) was added gradually over 30 min with continuous stirring at room temperature. Subsequently, 2 mL of a 6.25 M ethanolic solution of monochloroacetic acid was introduced, and the mixture was stirred at 60 °C for 5 h. The resulting solid was separated by filtration, washed with ethanol (5 × 5 mL), and dried under vacuum at 50 °C for 24 h. The obtained sodium salt of carboxymethyl starch (CMS-Na, 3 g) was dissolved in 100 mL of deionized water, and concentrated hydrochloric acid (approximately 3 mL) was added until the pH reached 2, followed by the gradual addition of ethanol to induce precipitation. The precipitate was filtered, washed with ethanol (5 × 5 mL), and dried under vacuum at 30 °C for 24 h.

#### 2.4.1 Determination of the Degree of Substitution with Carboxyl Groups (DS)

The degree of substitution of carboxymethyl starch (CMS-H) was determined by acid–base titration. The dried sample was ground to a fine powder, and 200 mg samples were weighed for analysis. Each sample was mixed with 20 mL of 0.05 M sodium hydroxide (NaOH) solution and stirred at room temperature for 30 min. Subsequently, the mixture was titrated with 0.06 M hydrochloric acid (HCl) using phenolphthalein as an indicator.^26^ All titrations were performed in triplicate. The degree of substitution was calculated using the following equation

2 $$DS=\frac{\left(162\times n_{COOH}\right)}{(m-\left(58\times n_{COOH}\right)}$$

where: *m* represents the mass of the CMS sample in grams, *n*_COOH_ is the number of moles of carboxyl groups calculated using the formula *n*_COOH_ = *V*_NaOH_ × *C*_NaOH_ – *V*_HCl_ × *C*_HCl_, where *C*_NaOH_ and *V*_NaOH_ are the concentration (mol/L) and volume (L) of the sodium hydroxide solution, and *C*_HCl_ and *V*_HCl_ are the concentration (mol/L) and volume (L) of the hydrochloric acid solution; the value 162 corresponds to the molar mass of a glucose unit in grams per mole, the value 58 represents the difference in product weight in grams.

### 2.5 Preparation of Carboxymethyl Starch Functionalized with 3-Aminophenylboronic Acid (CMS-PBA)

1-Ethyl-3-(3-(dimethylamino)­propyl)­carbodiimide (EDC, 10.0 mg; 0.06 mmol) was dissolved in 2 mL of deionized water. Separately, 1.0 g of carboxymethyl starch (CMS) was suspended in 20 mL of deionized water and combined with the EDC solution. After stirring at room temperature for 15 min, 3-aminophenylboronic acid (0.1 g; 0.73 mmol), dissolved in 3 mL of water, was added. The reaction mixture was stirred at room temperature for 24 h. The resulting solid was separated by filtration, washed with deionized water (3 × 5 mL), and dried under vacuum at 30 °C for 24 h.

### 2.6 Synthesis of Starch-Coated Magnetic Nanoparticles (S-MNPs)

The synthesis of magnetic nanoparticles coated with starch was carried out according to the procedure described in the literature.^28^ Corn starch (2.0 g) was dissolved in 100 mL of deionized water at 40 °C under stirring. Ferrous chloride tetrahydrate (1.14 g; 7.4 mmol) and ferric chloride hexahydrate (4.06 g; 15.0 mmol) were then added. A 30% aqueous sodium hydroxide solution (30 mL) was gradually added until the pH reached 13. The resulting black precipitate of starch-coated magnetic nanoparticles (S-MNPs) was separated using a magnet, washed with deionized water (5 × 10 mL), and dried under vacuum at 30 °C for 24 h.

### 2.7 Synthesis of Dialdehyde Starch-Coated Magnetic Nanoparticles (DAS-MNPs)

Starch-coated magnetic nanoparticles (1 g) were suspended in deionized water (10 mL), followed by the addition of an aqueous sodium periodate solution (0.70 M, 10 mL). The reaction vessel was immediately wrapped in aluminum foil to protect the oxidant from light-induced degradation. The mixture was stirred at 40 °C for 3 h. After cooling to room temperature, the oxidized magnetic nanoparticles were separated using a magnet, washed with deionized water (5 × 10 mL), and dried under vacuum at 30 °C for 24 h.

### 2.8 Synthesis of Dialdehyde Starch-Coated Magnetic Nanoparticles Functionalized with 3-Aminophenyl Boronic Acid (DAS-PBA-MNPs)

First, dialdehyde starch-coated magnetic nanoparticles (DAS-MNPs) were suspended in methanol (15 mL), followed by the addition of 3-aminophenylboronic acid (0.15 g, 1 mmol) dissolved in methanol (3 mL). The reaction mixture was stirred at room temperature for 24 h. Subsequently, a methanolic solution of sodium borohydride (0.03 M, 2 mL) was added, and stirring was continued for 6 h. The resulting nanoparticles were separated using a magnet, washed with methanol (5 × 5 mL), and dried under vacuum at 30 °C for 24 h.

### 2.9 Synthesis of Carboxymethyl Starch-Coated Magnetic Nanoparticles (CMS-MNPs)

Starch-coated magnetic nanoparticles (3 g) were suspended in ethanol (10 mL). Next, an aqueous sodium hydroxide solution (5 M, 2 mL) was added dropwise over 30 min at room temperature under stirring. Subsequently, an ethanolic solution of monochloroacetic acid (5 M, 2 mL) was added, and the mixture was heated to 60 °C and stirred for 5 h. After cooling to room temperature, the nanoparticles were separated using a magnet, washed with ethanol (5 × 5 mL), and dried under vacuum at 30 °C for 24 h.

### 2.10 Synthesis of Carboxymethyl Starch-Coated Magnetic Nanoparticles Functionalized with 3-Aminophenyl Boronic Acid (CMS-PBA-MNPs)

Sodium carboxymethyl starch-coated magnetic nanoparticles (CMS-MNPs, 2.50 g) were suspended in deionized water (20 mL). Then an aqueous solution of 1-ethyl-3-(3-(dimethylamino)­propyl)­carbodiimide (EDC, 10.0 mg; 0.06 mmol in 2 mL water) was added, and the mixture was stirred at room temperature for 15 min. A solution of 3-aminophenylboronic acid (0.1 g; 0.73 mmol) was introduced, and stirring was continued for 24 h at room temperature. The obtained nanoparticles were separated magnetically, washed with deionized water (5 × 5 mL), and dried under vacuum at 30 °C for 24 h.

### 2.11 Functional Accessibility of Boronic Acid Groups

#### 2.11.1 Calibration Curves for Alizarin Red S

Calibration curves for Alizarin Red S (ARS) were prepared separately in phosphate buffer (50 mM, pH 7.4) and carbonate buffer (50 mM, pH 9.0) in order to account for the pH-dependent absorbance of the dye. A stock solution of ARS (0.50 mM) was prepared in the corresponding buffer and subsequently diluted to obtain standard solutions with concentrations ranging from 0.01 to 0.25 mM.

The absorbance of each standard solution was measured at 520 nm using a UV–vis spectrophotometer. Linear calibration curves were constructed by plotting absorbance versus ARS concentration, and the resulting equations were used to determine ARS concentrations in binding experiments.^30^

#### 2.11.2 Assessment of Functional Accessibility of Boronic Acid Groups

The functional accessibility of boronic acid groups was evaluated by quantifying the amount of ARS bound to the materials after incubation. In a typical experiment, a defined mass of the tested material (10–50 mg) was dispersed in a known volume of buffer, and a defined volume of ARS solution with a known concentration was added. The suspensions were incubated at room temperature for 30 min under gentle shaking to allow the formation of boronate ester complexes between ARS and accessible boronic acid groups. The pH of the incubation medium was maintained at either 7.4 (phosphate buffer) or 9.0 (carbonate buffer) throughout the experiment. After incubation, the solid materials were separated from the liquid phase by magnetic decantation in the case of magnetic nanocomposites or by syringe microfiltration in the case of polymer powders. The absorbance of the supernatant was then measured at 520 nm. The concentration of ARS remaining in the supernatant was determined using the appropriate calibration curve corresponding to the buffer pH. The amount of ARS bound to the material was calculated as the difference between the initial amount of ARS added to the system and the amount remaining in the supernatant after incubation. The binding capacity was expressed as micromoles of bound ARS per gram of material (μmol g^–1^), which reflects the amount of functionally accessible boronic acid groups capable of forming boronate ester complexes under the applied conditions.

### 2.12 Investigation of the Binding Capacity of Glucose by the Obtained Materials

The amount of bound glucose was determined spectrophotometrically using the 3,5-dinitrosalicylic acid (DNS) and a UV–vis spectrophotometer (UV-1601 PC Shimadzu). Two different buffers were used: phosphate buffer (50 mM; pH 7.4) and carbonate buffer (50 mM; pH 9.0). For each buffer, a separate calibration curve for glucose was prepared.

#### 2.12.1 Glucose Standard Curve in Phosphate Buffer at pH 7.4 (50 mM)

First, a 100 mM glucose stock solution was prepared in 50 mM phosphate buffer (pH 7.4). Working solutions with concentrations of 10, 15, 20, 25, 30, and 35 mM were obtained by appropriately diluting the stock solution with the same buffer. A 1% (w/v) 3,5-dinitrosalicylic acid (DNS) reagent was prepared in 0.4 M NaOH. For each calibration point, 1 mL of glucose solution, 1 mL of DNS reagent, and 5 mL of deionized water were mixed. A blank sample was prepared using phosphate buffer instead of glucose solution. All samples were heated in a boiling water bath for 15 min with periodic mixing and then cooled to room temperature in a cold water bath. Absorbance was recorded at 540 nm, and the values were plotted against glucose concentration to generate a calibration curve. The same procedure was repeated using 50 mM carbonate buffer at pH 9.0.

#### 2.12.2 Quantitative Determination of Bound Glucose by the Obtained Materials in Phosphate Buffer at pH 7.4 (50 mM)

Modified polysaccharides (100–300 mg), including CMS-PBA and DAS-PBA, were incubated with 5 mL of glucose solution (29.85 mM in phosphate buffer, pH 7.4; 50 mM) in a thermomixer at 600 rpm for 15 min at room temperature. After a short settling period (∼5 min), 1 mL of the supernatant was withdrawn using a syringe fitted with a microfilter. The filtrate was then added 1 mL of a 1% (w/v) DNS reagent prepared in 0.40 M NaOH and 5 mL of deionized water. The mixtures were heated in a boiling water bath for 15 min with periodic manual mixing and then cooled to room temperature. A reference sample was prepared by treating the polysaccharide with phosphate buffer (without glucose) under the same conditions. Absorbance was measured at 540 nm. All experiments were performed in triplicate. The same procedure was repeated using 50 mM carbonate buffer at pH 9.0.

### 2.13 Investigation of the Binding Capacity of Glycoproteins by the Obtained Materials

The amount of bound α-1-acid glycoprotein was determined by emission spectroscopy in two different environments: in phosphate buffer at pH 7.4 and in carbonate buffer at pH 9.0. For this purpose, two appropriate calibration curves were created. The binding of α-1-acid glycoprotein was performed using a thermomixer. The amount of bound glycoprotein was determined using a spectrofluorimeter (JASCO FP-8300) with a temperature attachment.

#### 2.13.1 Calibration Curve of α-1-Acid Glycoprotein in Phosphate Buffer pH 7.4 (50 mM)

A stock solution of α-1-acid glycoprotein (10 μM) was prepared in phosphate buffer (50 mM, pH 7.4). A series of standard solutions with concentrations ranging from 1 to 10 μM was obtained by volumetric dilution of the stock solution with the same buffer. Fluorescence spectra of the solutions were recorded at 25 °C in the range of 300 to 400 nm with an excitation wavelength of 289 nm. The following parameters were applied: spectral registration range 300–400 nm, scanning speed 100 nm/min, spectral slit width Em/Ex 2.5 nm/5 nm. Three repetitions were performed. The same procedure was repeated using 50 mM carbonate buffer at pH 9.0.

#### 2.13.2 Binding of α-1-Acidic Glycoprotein by the Obtained Materials in Phosphate Buffer pH 7.4 (50 mM)

To assess the binding affinity of the tested materials (DAS-PBA, DAS-PBA-MNPs, CMS-PBA, CMS-PBA-MNPs), samples (50–100 mg) were incubated with 2 mL of α-1-acid glycoprotein (AGP) solution (6.65 μM in phosphate buffer, 50 mM, pH 7.4) at 36 °C for 15 min using a thermomixer (600 rpm). After incubation, the unbound AGP was separated from the solid phase by magnetic decantation (in the case of magnetic materials) or filtration through a syringe filter (for nonmagnetic samples). Reference samples were prepared analogously using phosphate buffer without AGP. Fluorescence spectra of the collected supernatants were recorded at 25 °C with excitation at 289 nm and emission monitored from 300 to 400 nm. The following parameters were used: excitation slit 5 nm, emission slit 2.5 nm, scan speed 100 nm/min. Each measurement was performed in triplicate. The procedure was repeated under identical conditions using carbonate buffer (50 mM, pH 9.0).

### 2.14 Investigation of the Binding Capacity of Non-glycoproteins by the Obtained Materials

Binding experiments with human serum albumin (HSA) were performed to evaluate nonspecific protein adsorption by the obtained materials. HSA was selected as a representative nonglycosylated protein and used as a control under conditions identical to those applied for glycoprotein binding experiments.

Briefly, a defined mass of the tested material (100 mg of polymeric samples or magnetic nanocomposites) was dispersed in 2.0 mL of a 6 μM HSA solution prepared in the appropriate buffer (phosphate buffer, 50 mM, pH 7.4, or carbonate buffer, 50 mM, pH 9.0). The samples were incubated under the same conditions as those used in the α-1-acid glycoprotein (AGP) binding assays to ensure direct comparability of the results. After incubation, the solid phase was separated from the liquid phase by magnetic decantation for magnetic nanocomposites or by syringe microfiltration for polymeric samples. The concentration of unbound HSA in the supernatant was determined spectrophotometrically. For quantitative analysis, separate calibration curves for HSA were prepared in phosphate buffer (pH 7.4) and carbonate buffer (pH 9.0). Standard HSA solutions with known concentrations were prepared in the corresponding buffers, and the amount of HSA bound to the materials was calculated analogously to the AGP binding experiments, based on the difference between the initial protein concentration and the concentration remaining in the supernatant after incubation.

The binding capacity was expressed as milligrams of bound protein per gram of material (mg g^–1^).

### 2.15 Release of α-1-Acid Glycoprotein from Magnetic Nanocomposites

Release experiments were conducted exclusively for magnetic nanocomposites to evaluate the reversibility of α-1-acid glycoprotein (AGP) binding mediated by boronic acid–diol interactions. AGP-loaded magnetic nanocomposites were first obtained by incubation under binding conditions as described above (carbonate buffer, 50 mM, pH 9.0).

After completion of the binding step, the magnetic nanocomposites were separated from the supernatant by applying an external magnetic field, and the supernatants were collected for control analysis. The recovered nanocomposites were then resuspended in phosphate buffer (50 mM, pH 6.5) to induce AGP release. The suspensions were incubated for 30 min at room temperature under gentle agitation.

Following incubation, the magnetic nanocomposites were separated again using an external magnetic field, and the supernatants containing the released AGP were collected. The concentration of released AGP was quantified using the same protein assay as applied in the binding experiments. For quantitative determination, a separate calibration curve for AGP was prepared in phosphate buffer (50 mM, pH 6.5).

The release efficiency was calculated as the percentage of released AGP relative to the amount of protein initially bound. All experiments were performed at least in duplicate.

## 3 Characterization of the Obtained Materials

### 3.1 NMR

Solid-state ^13^C NMR spectra of modified polysaccharides obtained were recorded on a Bruker 700 MHz NMR spectrometer, and ^11^B NMR spectra on a Bruker 400 MHz NMR spectrometer.

### 3.2 ATR–FTIR Spectroscopy

The ATR–FTIR spectra were recorded at room temperature using the spectrophotometer Spectrum Two (PerkinElmer, Waltham, MA, USA) equipped with a diamond crystal in the range of 4000–450 cm^–1^, resolution 16 cm^–1^, and 64 scans. Normalization and ATR correction of the spectra were performed.

### 3.3 Contact Angle Measurement

The contact angle (θ) of starch, DAS-PBA, and CMS-PBA was measured at 24 °C by the sessile drop method using an OCA 15 EC goniometer (DataPhysics Instruments). Glycerin and diiodomethane were used for the measurement. At least three measurements were performed to assess each sample, and the surface free energy (γs) was calculated by the standard Owens–Wendt method.

### 3.4 Scanning Electron Microscopy (SEM) and Transmission Electron Microscopy (TEM)

The morphology of boronic acid-functionalized polysaccharides and their corresponding polymer-coated magnetic nanoparticles was studied with a scanning electron microscope (1430 VP, LEO Electron Microscopy Ltd.). The samples were sputtered with gold before measurement. Transmission electron microscopy images with atomic resolution were taken using a FEI Europe microscope, model Tecnai F20 X-Twin.

### 3.5 Dynamic Light Scattering (DLS) and Potential Zeta Analysis

Dynamic light scattering (DLS) and Zeta potential measurements were performed using a Nano Zetasizer ZS90 instrument (Malvern, UK). DLS measurements were performed at a wavelength of 633 nm, using a detection angle at 25 °C. All measurements were triplicated, and the results were reported as hydrodynamic mean diameter values. Zeta potential measurement was measured in deionized water with the following parameters: (refractive index RI = 1.330, viscosity = 0.8872 cP, and dielectric constant = 78.5).

### 3.6 X-ray Diffraction (XRD)

XRD analysis used a Pro Philips X’PERT diffractometer (Cu Kα1, wavelength 1.54056 Å). Measurements were performed at room temperature over the 2θ range of 20–70°, with a step of 0.020°.

### 3.7 Thermogravimetric Analysis

Thermogravimetric analysis of the boronic acid-functionalized polysaccharides and their corresponding polymer-coated magnetic nanoparticles was performed on a TA Instruments (SDT 2960 Simultaneous DSC-TGA thermogravimetric analyzer) at a 10 °C/min heating rate in the range from ambient to 800 °C under a nitrogen atmosphere.

### 3.8 Sorption of Nitrogen

Nitrogen adsorption–desorption measurements at −196 °C were conducted using an ASAP 2020 Plus automated physisorption analyzer. The specific surface area of the samples was calculated based on the Brunauer–Emmett–Teller (BET) method. Pore size distribution was determined using the density functional theory (DFT) method.

### 3.9 Magnetization Study

Magnetization hysteresis loops of unmodified and starch-coated magnetic nanoparticles were recorded at 298 K. The measurements were carried out at the Laboratory of Magnetic Measurements, Faculty of Chemistry, University of Wrocław, Poland, using a superconducting quantum interference device (SQUID, MPMS3, San Diego, CA, USA).

## 4 Results and Discussion

Two starch derivatives-dialdehyde starch (DAS) and carboxymethyl starch (CMS)-were selected as polymer backbones for functionalization with 3-aminophenylboronic acid in order to investigate how differences in chemical structure influence the presentation and accessibility of boronic acid groups. The resulting materials, DAS-PBA and CMS-PBA, were designed to provide distinct polymer architectures while maintaining comparable boronic acid functionality.

Dialdehyde starch, obtained via periodate oxidation with an aldehyde content of 31%, was reacted with 3-aminophenylboronic acid to form a Schiff base, which was subsequently reduced to yield stable secondary amine linkages. This modification resulted in the DAS-PBA polymer, in which boronic acid moieties are anchored through reduced imine bridges. Owing to this mode of attachment, DAS-PBA can be considered a material with a relatively rigid polymer structure.

In parallel, carboxymethyl starch (CMS), synthesized by etherification with monochloroacetic acid (degree of substitution = 0.31), was functionalized with the same ligand via carbodiimide-mediated amidation using EDC. In this case, boronic acid groups were introduced through stable amide bonds, yielding the CMS-PBA polymer with enhanced chemical stability and a more flexible polymer backbone.

Comparison of the two systems highlights the impact of the linkage chemistry on polymer architecture. While both materials contain boronic acid functionalities, the different bonding modes-secondary amine linkages in DAS-PBA and amide linkages in CMS-PBA-are expected to influence polymer flexibility and, consequently, the functional accessibility of boronic acid groups.

As a further step, both polymers were employed as coatings for iron oxide nanoparticles to obtain hybrid magnetic systems. These nanocomposites combine the affinity properties of boronic acid-functionalized starch with the magnetic responsiveness of the nanoparticle core, enabling rapid separation of the solid phase and facilitating comparative studies between polymeric and magnetic systems. The synthetic pathways for both polymer modifications are shown schematically in Figures 1 and 2, illustrating the structural transformations responsible for the glycoprotein-binding functionality.

**Figure 1:**
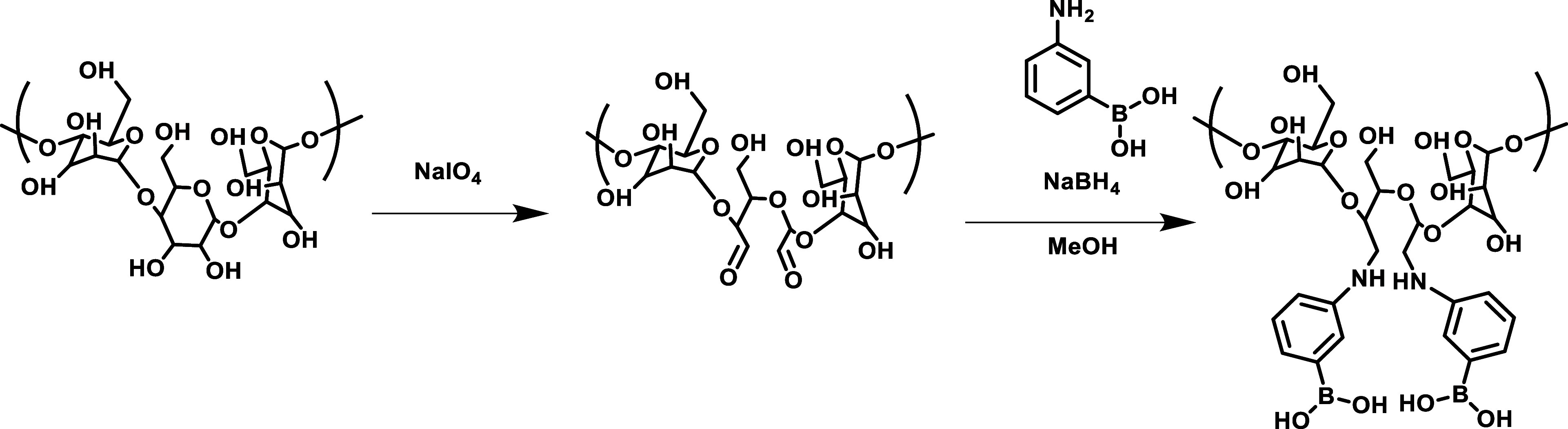
Scheme of starch modification leading to DAS-PBA.

**Figure 2:**
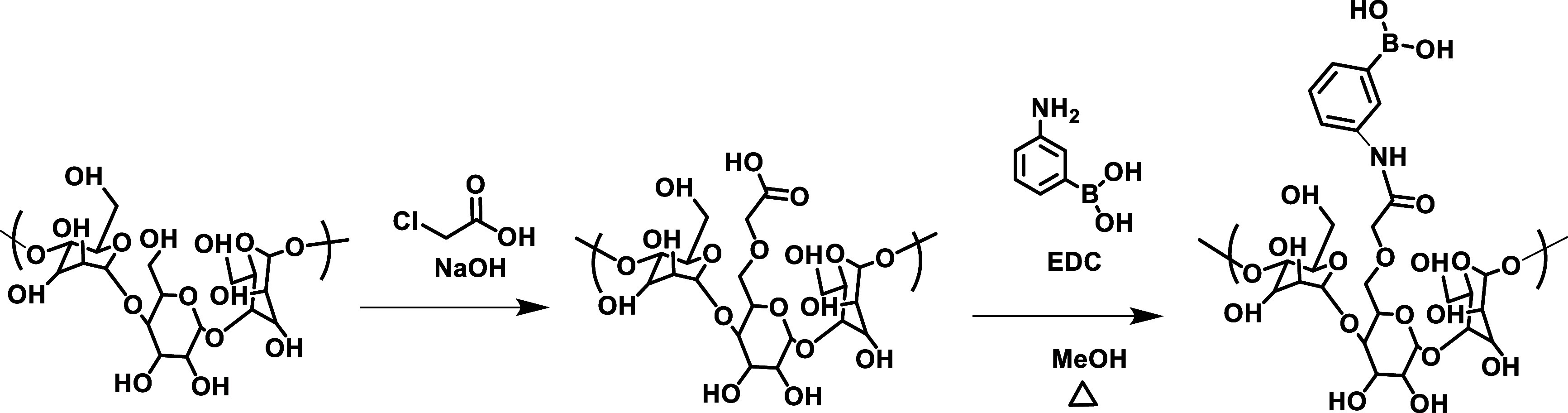
Scheme of starch modification leading to obtaining CMS-PBA.

## 5 Characterization of Materials

### 5.1 Characterization of Polysaccharides

#### 5.1.1 NMR

Solid-state NMR spectroscopy confirmed the structure of boron-functionalized starch-based materials. The ^13^C NMR spectra of native corn starch, dialdehyde starch modified with 3-aminophenyl boronic acid (DAS-PBA), and carboxymethyl starch modified with 3-aminophenyl boronic acid (CMS-PBA) are shown in Figure 3A, while the corresponding ^11^B NMR spectra are presented in Figure 3B. In the ^13^C NMR spectrum of native corn starch, characteristic signals appear at 61.5 ppm (C6), 72.7 ppm (C2, C3, C5), 82.5 ppm (C4), and 103.2 ppm (C1).^31^ The successful reaction between dialdehyde starch and 3-aminophenyl boronic acid was confirmed by the presence of a signal in the ^13^C NMR spectrum of the DAS-PBA at 130.1 ppm, attributed to the carbon atoms of the aromatic rings from the boronic acid. Additionally, the ^11^B NMR spectrum confirms the presence of boron atoms. The successful coupling of 3-aminophenyl boronic acid, mediated by EDC, is confirmed by a signal at 135.3 ppm, corresponding to the aromatic carbon atoms of the boronic acid involved in the reaction. Moreover, a signal at 173 ppm confirms the formation of an amide bond between the modified starch and boronic acid. In the ^11^B NMR spectrum, a signal at 6.93 ppm indicates boron’s presence in the CMS-PBA structure.

**Figure 3:**
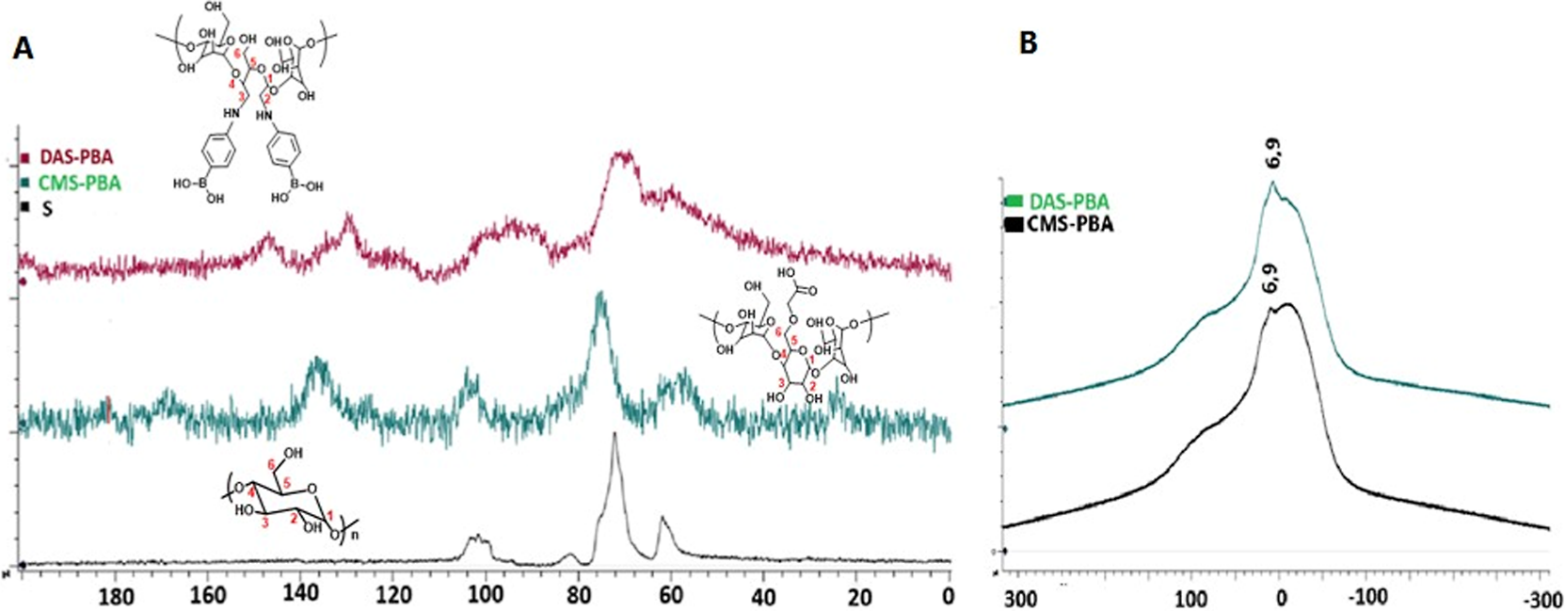
NMR spectra of the modified polysaccharides. Solid-state ^13^C NMR (A) and ^11^B NMR (B) spectra of S, DAS-PBA, and CMS-PBA.

#### 5.1.2 ATR–FTIR

ATR–FTIR spectroscopy revealed characteristic absorption bands corresponding to the functional groups present in the samples (Figure 4). A broad band centered around ∼3300 cm^–1^, observed in all spectra, is attributed to O–H stretching vibrations. Aliphatic C–H stretching modes appeared near 2880 cm^–1^, while bands at 1330 cm^–1^ and 1045 cm^–1^ correspond to C–H bending and C–O stretching vibrations within glucose units, respectively. The absorption at 1075 cm^–1^ is characteristic of C–O–C glycosidic linkages in the starch backbone.^32^

**Figure 4:**
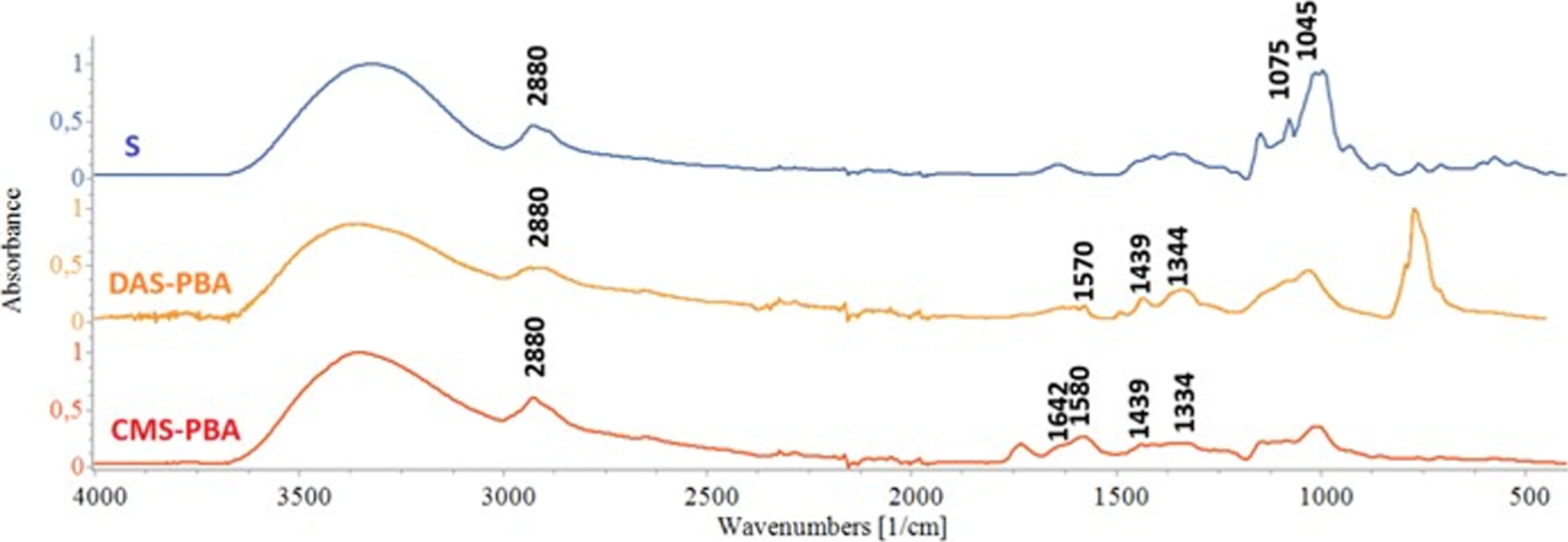
ATR–FTIR: starch (S), DAS-PBA and CMS-PBA.

A distinct band at 1570 cm^–1^ was observed in the spectrum of DAS-PBA, which was attributed to secondary amine formation resulting from reductive amination following NaBH_4_ treatment. Moreover, absorption bands at 1439 cm^–1^ and 1344 cm^–1^ correspond to B–C and B–O stretching vibrations, respectively, confirming successful incorporation of boronic acid moieties.^14^ The increased intensity and broadening of the hydroxyl band around 3300 cm^–1^ suggest enhanced hydrogen bonding, likely due to the presence of dihydroxyboronyl groups.

The spectrum of CMS-PBA showed a weak absorption band at 1733 cm^–1^, indicative of residual unreacted carboxylic groups, suggesting partial conversion during the amidation reaction. This observation is consistent with 13C NMR data, where residual carboxyl carbon signals overlap with resonances from newly formed amide carbons. Also, prominent bands at 1642 cm^–1^ and 1580 cm^–1^ were assigned to N–H bending and C–N stretching vibrations, respectively, further confirming successful amide bond formation.

#### 5.1.3 Contact Angle Measurments

The contact angle (θ) is a fundamental parameter for characterizing materials’ surface wettability and physicochemical properties. It describes the balance of adhesive forces between a liquid and a solid surface versus cohesive forces within the liquid.

This study employed the sessile drop method to measure the contact angles of two starch derivatives, DAS-PBA and CMS-PBA, using glycerine and diiodomethane as tested liquids. The Owens–Wendt method was then applied to calculate the total surface free energy and separate it into polar and dispersive components.^33,34^

The results (Table 1) reveal significant differences in surface properties due to the chemical modifications of starch. The DAS-PBA material exhibits low contact angles with both glycerol (43.2°) and diiodomethane (31.2°), corresponding to a high total surface free energy (51.46 mJ/m^2^). Notably, the polar component (18.69 mJ/m^2^) constitutes a substantial fraction of the total surface energy, indicative of pronounced surface polarity. This increased polarity can be attributed to the opening of the starch structure and the introduction of free dihydroxyboronyl groups, capable of forming hydrogen bonds and dipole interactions with polar liquids. These polar interactions facilitate increased wettability and suggest potential improvements in hydrophilicity, biocompatibility, and solubility in aqueous biological environments.

**Table 1:** Average Contact Angles of the Polymers with Test Liquids, along with the Values for Surface Free Energy and Its Components: Polar (γ_sp_) and Dispersive (γ_sd_)

	contact angles [θ, deg]				
	measuring liquid		surface free energy [mJ/m^2^]		
materials	glycerine	diiodomethane	γ_s_	γ_sd_	γ_sp_
S	59.2	44.7	45.02	27.32	17.69
DAS-PBA	43.2	31.2	51.46	32.77	18.68
CMS-PBA	88.9	58.6	28.92	27.46	1.46

Conversely, CMS-PBA shows markedly higher contact angles (88.9° with glycerol, 58.6° with diiodomethane), along with a significantly reduced total surface free energy (28.92 mJ/m^2^) and an almost negligible polar component (1.46 mJ/m^2^). The predominance of the dispersive component (27.46 mJ/m^2^) implies that van der Waals forces dominate the surface interactions. The hydrophobic nature of CMS-PBA is most likely caused by the presence of bulky aromatic groups and long polymer chains, which physically block or “hide” the hydrophilic groups on the material’s surface. As a result, these polar groups are less accessible for interactions with water or other polar liquids. These polar interactions contribute to increased wettability of the material, reflecting its altered surface chemistry.

In contrast, the chemical structure of DAS-PBA promotes stronger interactions with polar liquids. Its surface contains more accessible polar groups, which increases its overall surface polarity and makes it more hydrophilic. This also improves its potential to interact with biological molecules.

#### 5.1.4 Scanning Electron Microscopy

Scanning electron microscopy (SEM) was employed to investigate the surface morphology and structural alterations of the native starch and its chemically modified polymeric derivatives (Figure 5). The SEM images of native starch revealed granules with a predominantly spherical or ellipsoidal shape, exhibiting smooth and uniform surfaces. The granule sizes ranged from approximately 10 to 20 μm, consistent with typical features of plant-derived starch.

**Figure 5:**
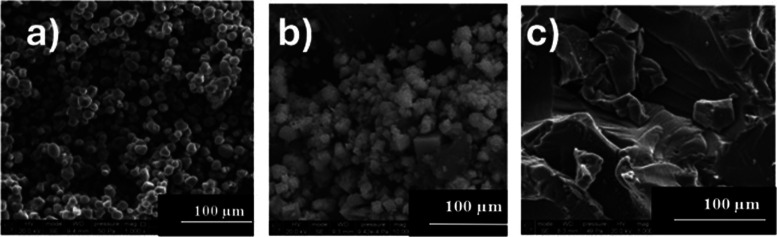
SEM images of (a) native starch, (b) DAS-PBA, (c) CMS-PBA.

Chemical modification of the polysaccharide induced significant morphological changes. The characteristic spherical architecture of the starch granules was disrupted, leading to partial or complete loss of their original shape. The modified materials displayed irregular and fragmented structures. These transformations are indicative of the breakdown and reorganization of the starch matrix during the functionalization process, suggesting the successful introduction of new functional groups and cross-linking events that alter the surface topology.

### 5.2 Characterization of Magnetic Nanoparticles

#### 5.2.1 ATR–FTIR

The ATR–FTIR spectra of magnetic nanoparticles functionalized with starch-based polymers, specifically dialdehyde starch-phenylboronic acid (DAS-PBA) and carboxymethyl starch-phenylboronic acid (CMS-PBA), presented all characteristic absorption bands corresponding to the functional groups present in the modified polysaccharide matrices (Figure 6). These included broad bands around 3300 cm^–1^, indicative of O–H stretching vibrations, as well as signals in the region of 1600–1000 cm^–1^, which are attributed to C=O stretching, C–O–C asymmetric stretching, and B–O vibrations from the phenylboronic acid moieties, confirming successful functionalization of the starch backbone. In addition to these polysaccharide-related features, a distinct absorption band was observed around 550 cm^–1^, which is attributed to the Fe^2+^–O^2–^ and Fe^3+^–O^2–^ stretching vibrations. This band is a signature of the spinel structure of magnetite (Fe_3_O_4_), thereby confirming the successful incorporation of a magnetic core within the synthesized nanostructures.^35^

**Figure 6:**
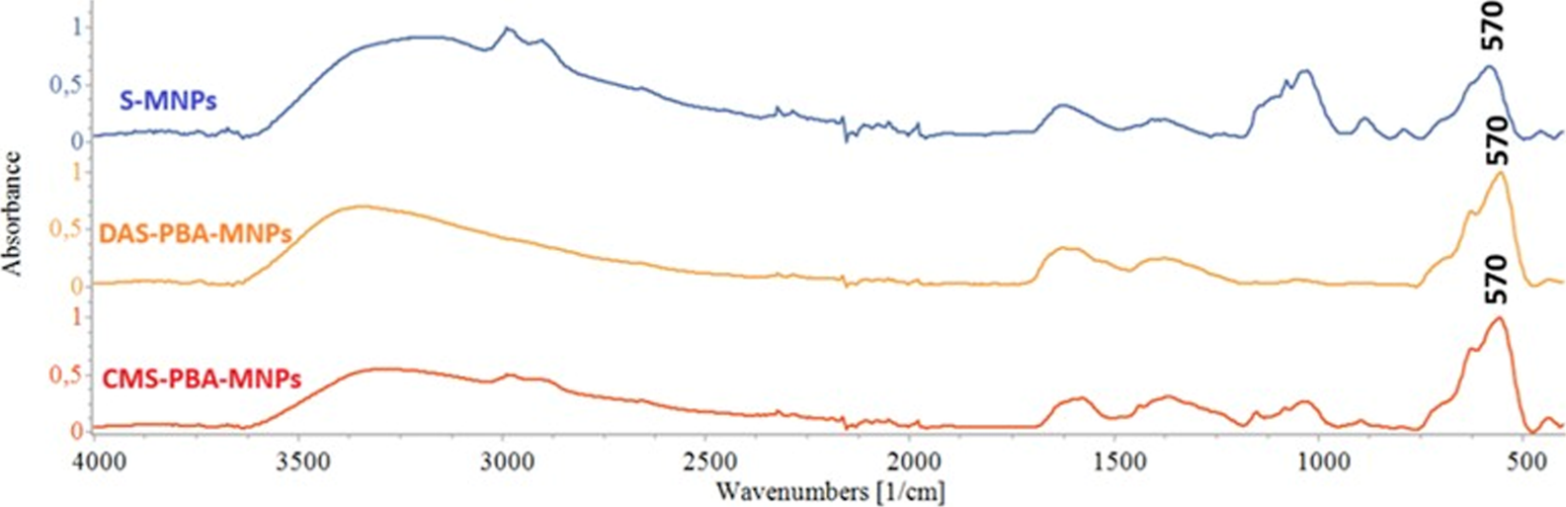
ATR–FTIR of S-MNPs, DAS-PBA-MNPs and CMS-PBA-MNPs.

#### 5.2.2 SEM

Furthermore, SEM analysis was conducted on magnetic nanoparticles functionalized with chemically modified polymers (Figure 7). The micrographs revealed a tendency of the nanoparticles to form agglomerates, which is characteristic behavior attributed to their high surface energy and intrinsic magnetic interactions. Nevertheless, the presence of the polymeric coating markedly influenced both the surface morphology and the extent of agglomeration. The modified polymers formed a distinct, continuous shell around the magnetic cores, resulting in nanostructures with relatively smoother and more defined contours than their uncoated counterparts. The coated nanoparticles exhibited an irregular yet discernible morphology, distinct from the unmodified polymer matrix and native starch. This indicates successful surface functionalization and effective integration of polymer and magnetic components.

**Figure 7:**
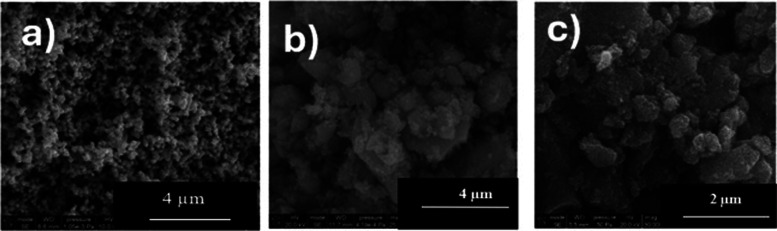
SEM images of (a) Fe_3_O_4_-MNPs, (b) DAS-PBA-MNPs, (c) CMS-PBA-MNPs.

#### 5.2.3 Transmission Electron Microscopy, X-ray Diffraction (XRD) and Dynamic Light Scattering (DLS) of Nanoparticles

Transmission electron microscopy (TEM), X-ray diffraction (XRD) and dynamic light scattering (DLS) analyses were employed to comprehensively characterize the synthesized magnetic nanoparticles (Figures 8 and 9). TEM imaging revealed that the nanoparticles exhibited predominantly irregular, hemispherical morphologies and showed a marked tendency to form aggregates under the observation conditions, which can be attributed to their inherent magnetic interactions. The average hydrodynamic diameter of the unmodified Fe_3_O_4_ nanoparticles, as determined by DLS, was approximately 15 nm. Upon surface modification with polymeric layers, the average diameters increased to 35 nm for DAS-PBA-MNPs and 36 nm for CMS-PBA-MNPs, respectively (Table 2). This measurable increase in particle size confirms the successful formation of a polymeric shell surrounding the magnetic cores. It should be noted that particle sizes determined by dynamic light scattering (DLS) represent hydrodynamic diameters measured in dispersion and therefore include not only the inorganic magnetite core but also the surrounding polymer shell and associated solvation layer. In contrast, TEM images depict dehydrated particles under high-vacuum conditions, where the polymer coating may partially collapse or become less distinguishable, and particle aggregation can occur during sample preparation. Consequently, the smaller particle sizes observed in TEM compared to DLS measurements are expected for polymer-coated magnetic nanoparticles and do not indicate discrepancies between the applied characterization techniques.^36–38^ Zeta potential measurements further supported the effective surface modification. The native starch displayed a strongly negative zeta potential of −33.9 mV,^39^ which significantly decreased upon polymer modification to −1.19 mV for DAS-PBA and −9.91 mV for CMS-PBA. Similarly, a reduction in surface charge was observed for the magnetic systems: while unmodified Fe_3_O_4_ nanoparticles presented a zeta potential of −18.2 mV,^39^ the modified particles showed a less negative surface charge (Table 2). These trends are in agreement with literature reports describing the impact of surface functionalization on the electrokinetic properties of magnetic nanomaterials.^40,41^

**Figure 8:**
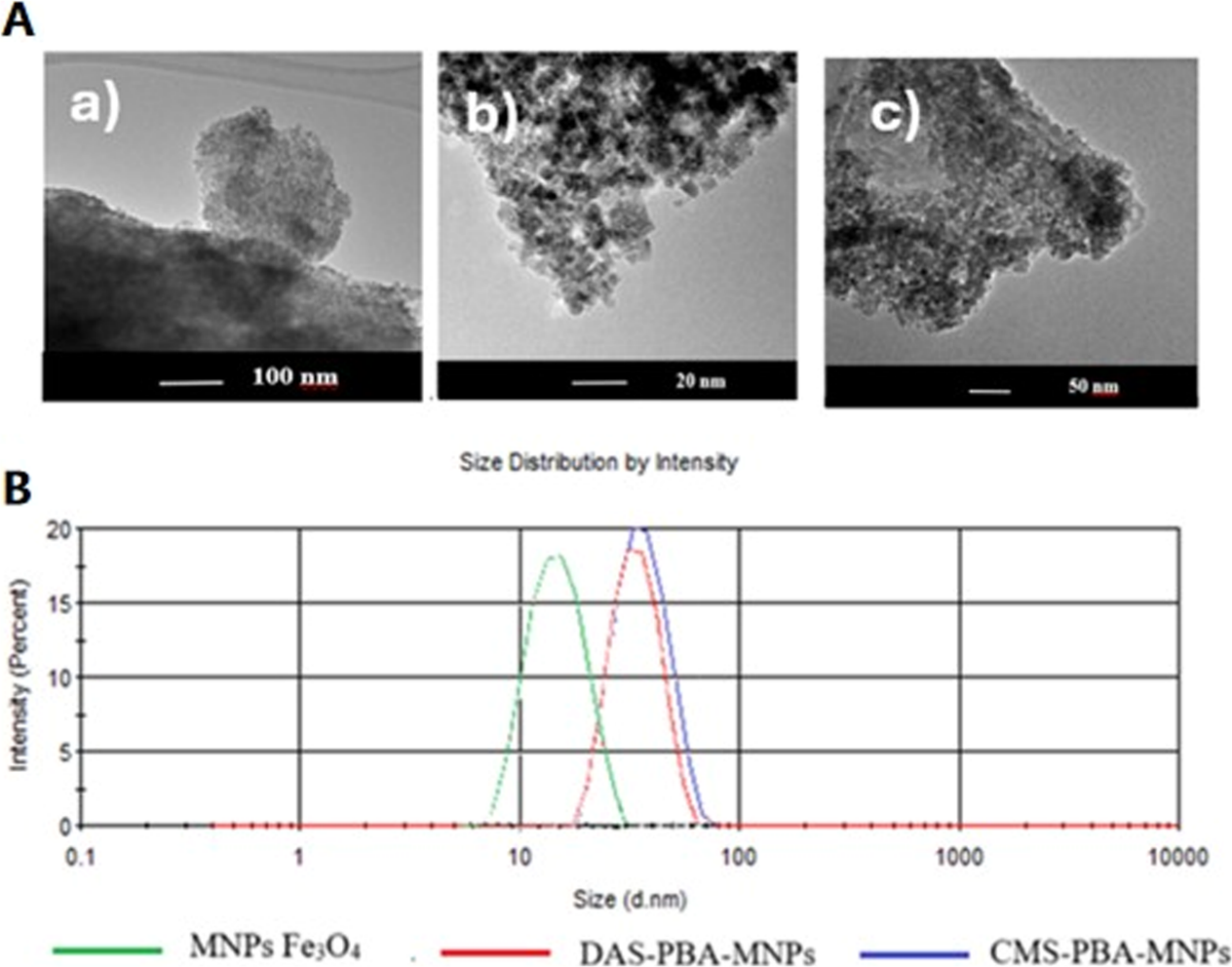
TEM images of (a) Fe_3_O_4_-MNPs (b) DAS-PBA-MNPs (c) CMS-PBA-MNPs (A); nanosize histogram of Fe_3_O_4_-MNPs, DAS-PBA-MNPs, CMS-PBA-MNP (B).

**Figure 9:**
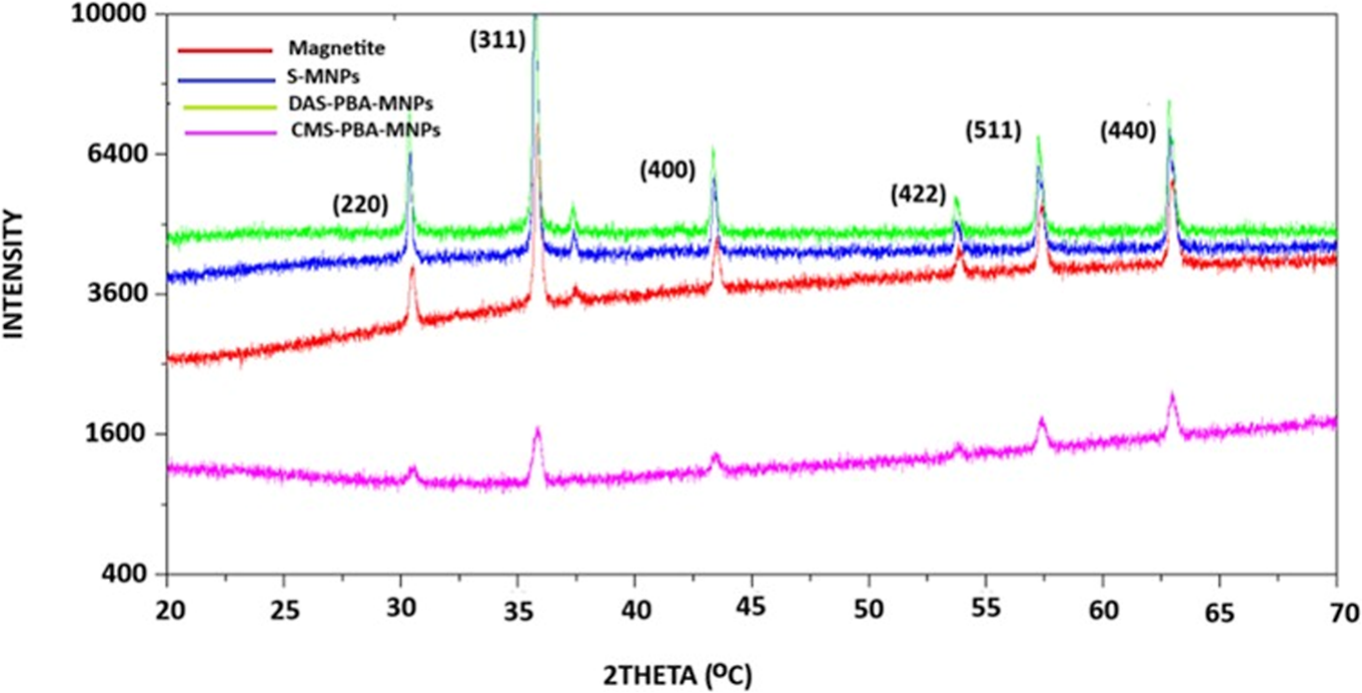
X-ray diffraction patterns of pure magnetite nanoparticles, magnetite nanoparticles with starch, CMS-PBA and DAS-PBA.

**Table 2:** Average Size and Potential Zeta Data

materials	size [nm]	potential zeta [mV]
DAS-PBA-MNPs	35	–17.4
CMS-PBA-MNPS	36	–16.7

X-ray diffraction (XRD) analysis was conducted to confirm the crystallographic structure of the magnetic core (Figure 9). The diffractograms of all nanoparticle formulations displayed six distinct peaks corresponding to the (220), (311), (400), (422), (511), and (440) crystallographic planes, located at 2θ values of approximately 30°, 35°, 43°, 53°, 57°, and 62°, respectively. These diffraction peaks match the characteristic pattern of magnetite (Fe_3_O_4_) and are consistent with standard reference data from the X’Pert HighScore database,^35–37^ thereby confirming the presence of a pure magnetite core in the synthesized nanostructures.

#### 5.2.4 Magnetic Characterization of Starch-Based Nanoparticles

The magnetic behavior of the synthesized nanoparticles was evaluated using vibrating sample magnetometry (VSM). The magnetization curves (*M*–*H*) recorded at room temperature revealed significant differences between unmodified Fe_3_O_4_ nanoparticles and their starch-based derivatives (Figure 10). The saturation magnetization (*M*_s_) of the bare Fe_3_O_4_ nanoparticles reached approximately 90 emu/g, consistent with values reported in the literature for bulk magnetite.^42,43^

**Figure 10:**
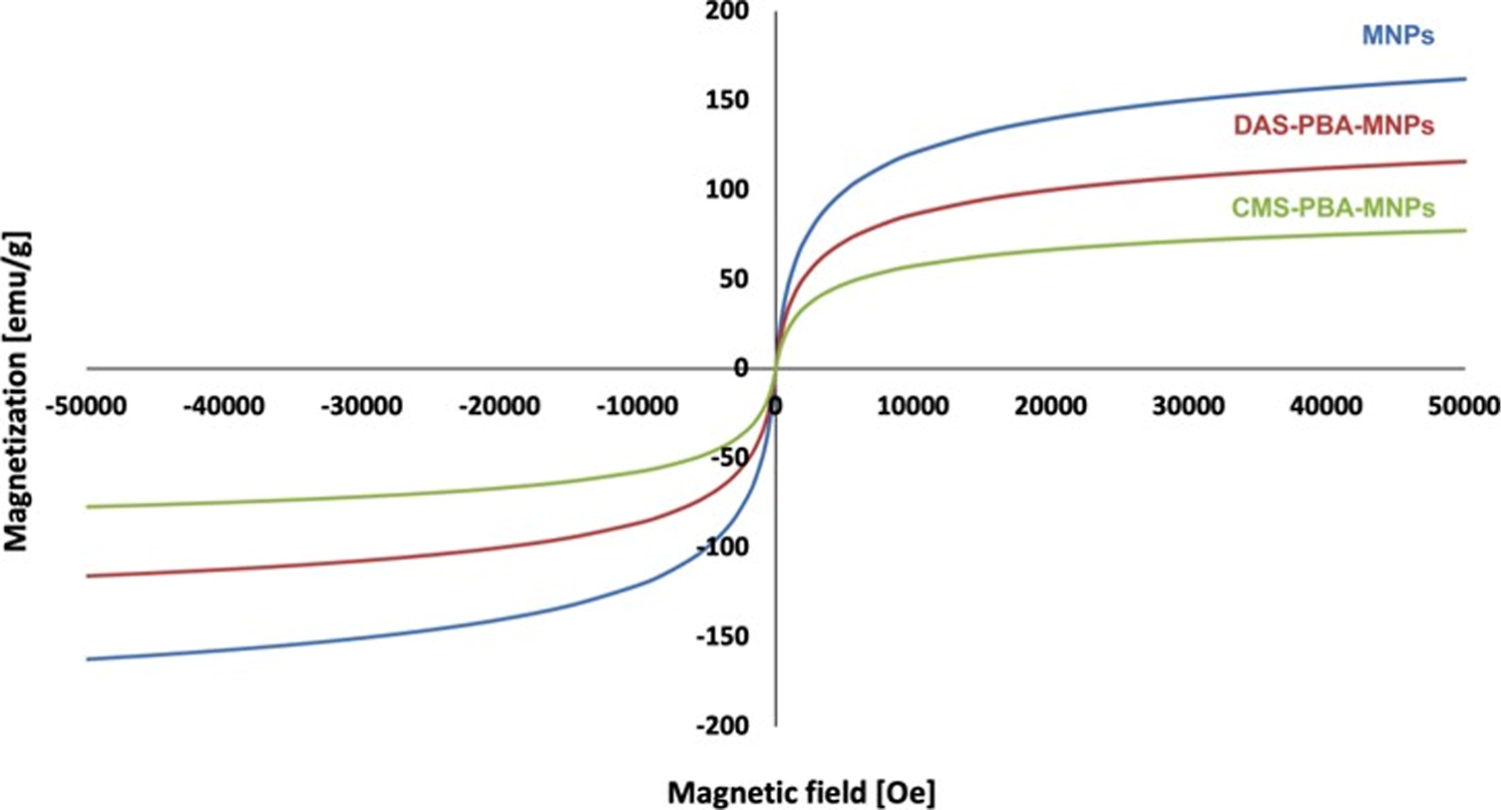
Magnetization curves of uncoated nanoparticles (MNPs) and starch-based magnetic nanocomposites (DAS-PBA-MNPs and CMS-PBA-MNPs) measured at room temperature.

A marked decrease in magnetic response was observed upon surface functionalization with starch derivatives. For DAS-PBA-MNPs, the *M*_s_ value dropped to approximately 60 emu/g, while for CMS-PBA-MNPs it further decreased to ∼50 emu/g. This reduction is attributed to a nonmagnetic organic coating on the magnetite core, which effectively dilutes the magnetic phase and suppresses the total magnetization per unit mass.

Interestingly, the CMS-PBA-MNPs exhibited lower magnetization than DAS-PBA-MNPs, which may be explained by their higher degree of functionalization and the more extensive hydration of the carboxymethylated polymer layer. The denser and more hydrophilic CMS matrix likely introduces a thicker organic shell around the magnetic core, further attenuating the material’s magnetic signal. Additionally, the increased steric hindrance and potential for electrostatic interactions in CMS-based coatings may alter the distribution or mobility of the surface spins, contributing to the overall suppression of magnetic behavior.

Despite the reduction in saturation magnetization, both DAS- and CMS-functionalized nanoparticles retained superparamagnetic-like behavior, with negligible remanence and coercivity, indicating their suitability for magnetically assisted separation under an external field. This combination of selective glycoprotein-binding capability with sufficient magnetic responsiveness supports the potential of these materials for use in clinically relevant diagnostic and bioanalytical platforms.

### 5.3 Thermogravimetric Analysis (TGA–DTA)

Thermogravimetric analysis (TGA) measures mass changes in materials with temperature or time in a controlled atmosphere, revealing thermal stability and decomposition behavior. This is vital for assessing new materials, especially for their protein-binding properties.^44^

The initial weight loss, approximately 11%, corresponds to the evaporation of adsorbed and bound water. The primary degradation occurs around 290 °C (*T*_max_ = 294 °C), resulting in a substantial mass loss of nearly 80%.

Among the modified samples, DAS-PBA presented the highest *T*_max_ at 340 °C. This indicates that chemical modification of starch significantly enhances its thermal stability. Specifically, the *T*_max_ increases by 46 °C for DAS-PBA and 57 °C for CMS-PBA compared to the native starch. This improvement is likely due to incorporating boron–oxygen (B–O) bonds within the polymer matrix. Given the high bond dissociation energy of B–O linkages, their incorporation may contribute significantly to the enhanced thermal stability of the modified materials (Figure 11A and Table 3).^39^

**Table 3:** Thermal Parameters of Materials: Starch, DAS-PBA, CMS-PBA, S-MNPs, DAS-PBA-MNPs, CMS-PBA-MNPs Determined from TGA–DTA Curves in a Nitrogen Atmosphere

	I step		II step			III step			IV step			
materials	*T*_max_ (°C)	Δ*m* (%)	*T*_o_ (°C)	*T*_max_ (°C)	Δ*m* (%)	*T*_o_ (°C)	*T*_max_ (°C)	Δ*m* (%)	*T*_o_ (°C)	*T*_max_ (°C)	Δ*m* (%)	residue 800 °C
S	56	11	290	294	80	-	-	-	-	-	-	9
DAS-PBA	47	7	285	340	27	286	301	28	-	-	-	38
CMS-PBA	70	7	269	311	56	-	-	-	-	-	-	37
S-MNPs	69	7	203	236	17	472	680	5	-	-	-	71
DAS-PBA MNPs	66	5	201	223	8	251	284	8	456	604	4	75
CMS-PBA-MNPs	63	4	223	266	11	472	666	6	712	756	3	76

A three-step thermal degradation profile was observed for the S-MNPs. The first step occurred at 20–160 °C (*T*_max_ = 69 °C), corresponding to approximately 7% mass loss. The second step, observed between 203 and 420 °C with a mass loss of about 17% is attributed to the thermal decomposition of the organic starch coating. The third stage, with *T*_max_ above 600 °C, is associated with structural changes within the inorganic magnetite core.

Magnetic nanoparticles coated with DAS-PBA presented thermal stability comparable to that of those with unmodified starch. In contrast, CMS-PBA-MNPs demonstrate superior thermal resistance relative to DAS-PBA MNPs and S-MNPs (Figure 11B). Notably, the fourth degradation stage in all samples occurred above 500 °C and is associated with transformations within the inorganic core. At this temperature, magnetite to undergoes phase transitions to maghemite (Fe_2_O_3_) or nonstoichiometric iron oxide Fe_1–*x*_O^15,45,46^ Although this transformation typically requires an oxidizing environment like air, decomposition products from the starch-based material likely contribute oxygen species that facilitate oxidation even under otherwise inert or limited-oxygen conditions.^47^

**Figure 11:**
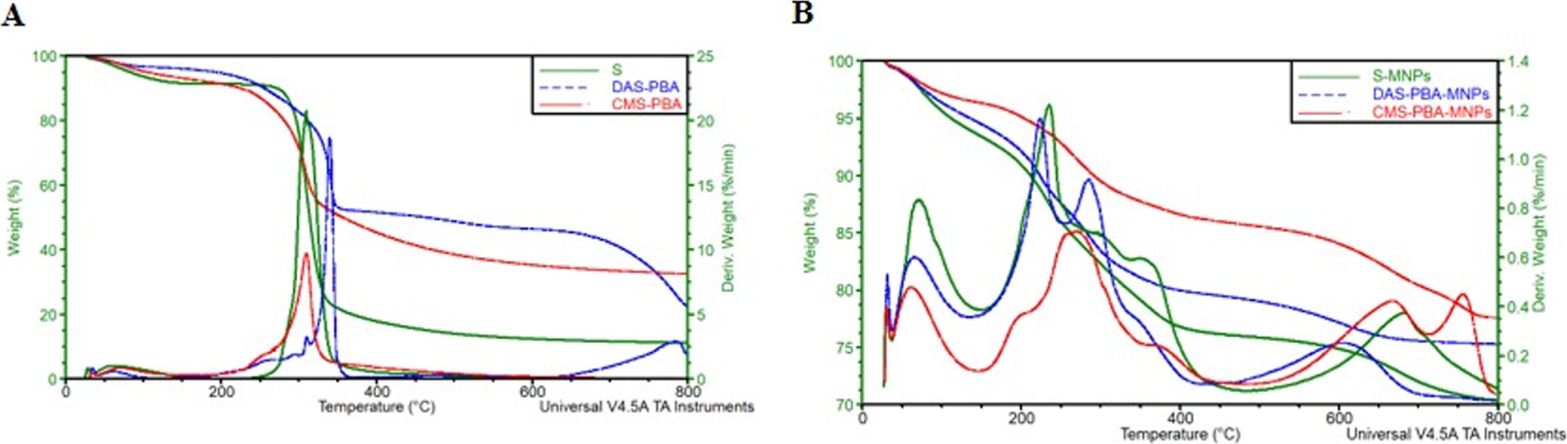
Thermogravimetric curve for starch, DAS-PBA, CMS-PBA (A), S-MNPs, DAS-PBA-MNPs, CMS-PBA-MNPs (B).

### 5.4 Brunauer–Emmett–Teller Method

The specific surface areas of DAS-PBA and CMS-PBA samples, determined using the Brunauer–Emmett–Teller (BET) method, were found to be 0.5 m^2^/g and 20.0 m^2^/g, respectively (Figure 12). These results indicate that the presence of carboxymethyl substituents in CMS-PBA significantly enhances the surface area compared to DAS-PBA. Upon modification with Fe_3_O_4_ nanoparticles, a substantial increase in surface area was observed. Specifically, the BET surface areas of DAS-PBA-MNPs and CMS-PBA-MNPs were measured to be 102 m^2^/g and 91 m^2^/g, respectively. These findings suggest that the deposition of magnetic nanoparticles markedly increases the specific surface area of the hybrid materials. Moreover, the unmodified starch derivative with free dihydroxyboryl groups exhibited a nonporous structure, as indicated by its negligible surface area.

**Figure 12:**
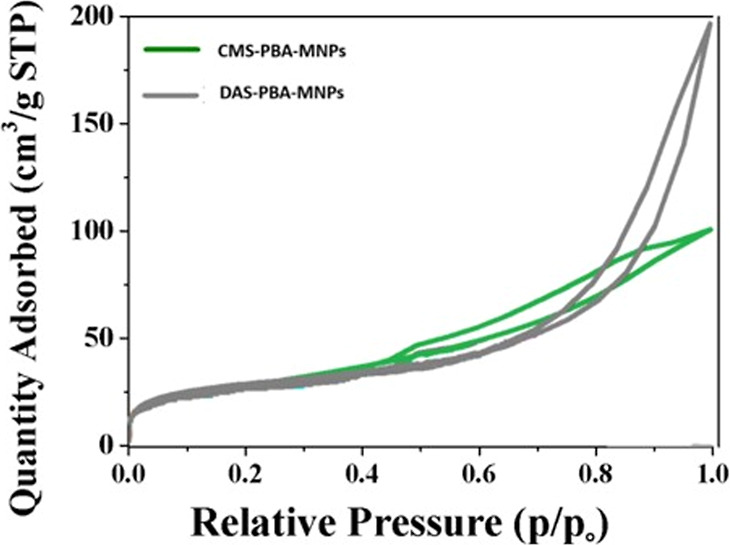
Nitrogen adsorption–desorption isotherms of CMS-PBA-MNPs and DAS-PBA-MNPs.

### 5.5 Assessment of Functional Accessibility of Boronic Acid Groups

The accessibility of boronic acid groups was investigated using Alizarin Red S (ARS), a molecule that forms reversible boronate ester complexes with *cis*-diol-containing ligands. This distinction is particularly important for starch-based systems, as starch is a diol-rich polysaccharide and intra- or intermolecular boronate ester formation between boronic acid groups and the polymer backbone may occur. Such internal interactions can reduce the number of boronic acid moieties available for binding external diol-containing ligands and therefore affect the apparent binding performance of the material. This approach enables the assessment of functionally accessible dihydroksyboryl groups rather than the total chemical boron content of the material. It should be emphasized that the ARS assay provides a functional measure of boronic acid accessibility under the applied conditions and does not quantify the total amount of boronic acid incorporated into the polymer matrix.

For all investigated materials, ARS binding exhibited a pronounced pH dependence. Significantly higher ARS binding was observed at pH 9.0 compared to pH 7.4, which is consistent with the formation of boronate anions under alkaline conditions. These anionic species are known to display enhanced affinity toward diol-containing molecules.

A clear difference in ARS binding capacity was observed between the polymeric materials and their corresponding magnetic nanocomposites (Table 4). The starch-based polymers functionalized with phenylboronic acid exhibited higher ARS binding values, indicating a larger fraction of accessible boronic acid groups. In contrast, the magnetic nanocomposites showed reduced ARS binding capacity. This decrease can be attributed to conformational constraints imposed by surface attachment.

**Table 4:** Functional Availability of Boronic Acid Groups

	functional accessibility of boronic acid groups (μmol g–1)	
materials	phosphate buffer pH 7.4 (50 mM)	carbonate buffer pH 9.0 (50 mM)
DAS-PBA	1.27 ± 0.03	9.23 ± 0.06
CMS-PBA	1.84 ± 0.05	14.01 ± 0.03
DAS-PBA-MNPs	0.35 ± 0.02	2.93 ± 0.12
CMS-PBA-MNPS	0.61 ± 0.03	5.97 ± 0.06

Comparison of the two polymer architectures further revealed that ARS binding capacity depended on the type of starch derivative used. These differences are likely related to variations in polymer flexibility and the spatial distribution of functional groups within the polymer matrix. Overall, the ARS assay demonstrates that phenylboronic acid groups remain functionally accessible in both polymeric and magnetic systems and retain their pH-responsive binding behavior. These findings support the suitability of the developed materials for further investigations into boronic acid–mediated interactions with diol-containing molecules, including glycoproteins.

The differences in functional accessibility of boronic acid groups between CMS-PBA and DAS-PBA can be attributed to the distinct polymer modification chemistries and backbone architectures. Carboxymethylation introduces negatively charged carboxyl groups along the starch chains, which promote electrostatic repulsion, enhanced hydration, and chain expansion in aqueous media, leading to a less densely packed and more open polymer architecture.^48^ These effects can reduce intramolecular interactions within the polymer matrix and limit internal boronate ester formation with the diol-rich backbone, resulting in improved exposure of B­(OH)_2_ groups.

Although dialdehyde starch promotes network stiffening through covalent inter- and intramolecular interactions, including local cross-linking and partial chain folding that increase polymer packing density and restrict overall chain mobility, this process does not result in complete consumption of reactive sites.^49^ Consequently, a heterogeneous polymer network is formed in which sufficient functional sites remain available for effective postfunctionalization with boronic acid moieties. The successful attachment and persistence of accessible B­(OH)_2_ groups are experimentally confirmed by ARS binding, demonstrating that the reduced binding performance of DAS-PBA arises from limited functional accessibility within a more compact polymer architecture rather than from the absence of boronic acid functionalities.

### 5.6 Glucose Binding to the Boronic Acid Functionalized Polymers Surface

The boronic acid-functionalized polymers DAS-PBA and CMS-PBA exhibited the ability to bind glucose through the formation of cyclic boronate ester complexes with the sugar’s *cis*-diol groups. The observed differences in glucose binding between CMS-PBA and DAS-PBA correlate directly with the functional accessibility of boronic acid groups determined by the ARS assay, underscoring the decisive role of polymer backbone architecture rather than the total boronic acid content.

The amount of glucose bound was strongly influenced by the pH of the medium, with significantly higher binding observed in carbonate buffer (pH 9.0) compared to phosphate buffer (pH 7.4). This behavior is consistent with the well-established pH-dependent chemistry of boronic acids, which form more stable boronate ester complexes with diols under alkaline conditions.^50,51^

Among the two materials, CMS-PBA exhibited a significantly higher glucose-binding capacity (186.2 ± 2.05 mg g^–1^ at pH 7.4 and 208.0 ± 2.37 mg g^–1^ at pH 9.0) compared to DAS-PBA, which bound 30.25 ± 1.02 mg g^–1^ and 42.55 ± 1.10 mg g^–1^, respectively. These pronounced differences correlate with the experimentally determined differences in the functional accessibility of boronic acid groups, as revealed by the ARS assay. CMS-PBA, based on carboxymethyl starch, exhibits a higher fraction of functionally accessible B­(OH)_2_ groups, which facilitates more efficient glucose binding (Table 5).

**Table 5:** Amount of Glucose Bound by the Polymer

	the amount of glucose bound by the polymer	
polymers	phosphate buffer pH 7.4 (50 mM) [mg/g]	carbonate buffer pH 9.0 (50 mM) [mg/g]
DAS-PBA	30.25 ± 1.02	42.55 ± 1.10
CMS-PBA	186.20 ± 2.05	208.00 ± 2.37

In contrast, the lower glucose binding observed for DAS-PBA can be attributed to the more compact and mechanically rigid dialdehyde-derived polymer network, characterized by higher packing density and restricted chain mobility, which limits effective boronic acid accessibility through partial intramolecular shielding within the diol-rich matrix. These results demonstrate that polymer network density and backbone flexibility are key determinants of boronic acid accessibility and glucose binding efficiency in starch-based materials. Similar structure–function relationships have been widely reported for boronate affinity systems, where effective binding toward *cis*-diol-containing ligands depends not only on the total boronic acid content but also on polymer architecture and local microenvironment effects^52,53^

### 5.7 Protein Binding Behavior of Boronic Acid-Functionalized Materials

Following the confirmation of glucose binding, the interaction of the obtained materials with proteins was investigated. The binding of α-1-acid glycoprotein (AGP), a highly glycosylated plasma protein, was evaluated and compared with that of human serum albumin (HSA), a nonglycosylated protein used to assess nonspecific protein adsorption (Figure 13).

**Figure 13:**
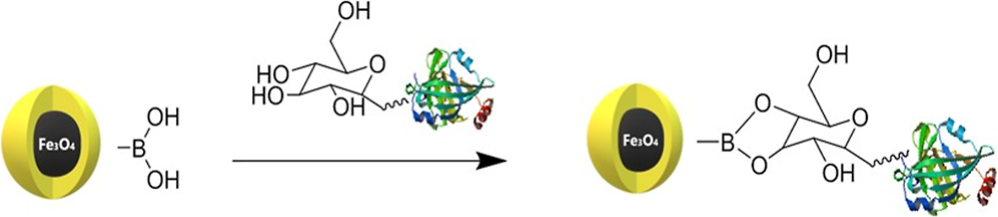
Schematic illustration of glycoprotein binding by magnetic nanoparticles coated with modified polymers.

Similarly to glucose binding, AGP binding was strongly pH-dependent. Both DAS-PBA and CMS-PBA exhibited enhanced AGP binding in carbonate buffer at pH 9.0 compared to phosphate buffer at pH 7.4, which is consistent with the increased reactivity of boronic acid groups under alkaline conditions and the formation of boronate ester interactions with *cis*-diol-containing glycans.

Among the investigated materials, CMS-PBA demonstrated superior AGP binding capacities (38.98 mg g^–1^ at pH 7.4 and 46.20 mg g^–1^ at pH 9.0). The obtained results are presented in Table 6. This behavior correlates with the higher functional accessibility of boronic acid groups in CMS-PBA, as independently confirmed by the ARS assay. The carboxymethyl starch backbone likely provides a more open and flexible polymer architecture, enabling effective presentation of B­(OH)_2_ groups for interaction with glycan moieties on AGP. In contrast, DAS-PBA exhibited significantly lower AGP binding, which is attributed primarily to reduced functional accessibility of boronic acid groups resulting from structural constraints of the dialdehyde-derived polymer backbone.

**Table 6:** Amount of Glycoprotein and Non-glicoprotein Bound by Materials

	the amount of α-1-acid glycoprotein bound by the material				
	phosphate buffer pH 7.4 (50 mM) [mg/g]		carbonate buffer pH 9.0 (50 mM) [mg/g]		
materials	AGP	HSA	AGP	HSA	release of bound AGP [%]
DAS-PBA	19.08 ± 0.57	0.74 ± 0.03	25.40 ± 0.42	0.81 ± 0.09	-
CMS-PBA	38.98 ± 0.42	0.67 ± 0.06	46.20 ± 0.38	0.94 ± 0.06	-
DAS-PBA-MNPs	7.50 ± 0.10	1.13 ± 0.09	10.89 ± 0.21	1.26 ± 0.04	9.57 ± 0.47
CMS-PBA-MNPs	18.29 ± 0.18	1.01 ± 0.11	21.30 ± 0.19	0.93 ± 0.02	12.67 ± 0.09

In contrast, DAS-PBA exhibited significantly lower AGP binding, which can be attributed to the more compact and mechanically rigid dialdehyde-derived polymer network. Aldehyde-mediated inter- and intramolecular interactions increase polymer packing density and restrict chain mobility, leading to partial intramolecular shielding of B­(OH)_2_ groups and reduced functional accessibility for glycoprotein binding. Such effects are consistent with broader reports describing how polymer rigidity, cross-linking density, and hydration influence boronic acid accessibility and multivalent binding behavior in affinity systems.^52,53^

Protein binding experiments performed with magnetic nanocomposites revealed lower AGP binding capacities compared to the corresponding free polymers, despite the advantages of rapid magnetic separation. This reduction is attributed to the partial masking of boronic acid groups upon coating on the magnetite surface. Electrostatic effects may further contribute to reduced binding efficiency but are considered secondary to functional group accessibility. In contrast to AGP, only negligible amounts of HSA were bound to both polymeric and magnetic materials (Table 6). This observation indicates that protein uptake observed for AGP is not dominated by nonspecific protein adsorption, but is primarily associated with boronic acid–glycan interactions. The low levels of HSA detected can be attributed to weak, nonspecific interactions with the material surface rather than affinity-driven binding. To further confirm that protein binding originates from boronic acid functionalities, control experiments were performed using PBA-free starch-based materials under analogous experimental conditions. No measurable binding of glucose or α-1-acid glycoprotein was observed for the unmodified materials, with values close to the detection limit. These results confirm that the observed protein uptake is not associated with the starch backbone itself, but arises from the presence of phenylboronic acid groups capable of forming boronate ester interactions with glycan moieties.

Overall, CMS-PBA exhibited the most favorable protein-binding performance due to the combined effects of polymer architecture and higher functional accessibility of boronic acid groups. While magnetic nanocomposites offer practical advantages in terms of separation and handling, their reduced binding capacities reflect structural and electrostatic limitations rather than diminished chemical functionality.

### 5.8 Release of α-1-Acid Glycoprotein from Magnetic Nanocomposites

Release experiments were conducted exclusively for the magnetic nanocomposites in order to demonstrate the reversibility of AGP binding in systems offering practical advantages in separation and handling. The application of an external magnetic field enables rapid and reproducible isolation of the solid phase, which is particularly advantageous for release studies where precise control of incubation time and buffer exchange is required. In contrast, polymer powders require additional separation steps and longer sedimentation times, making them less suitable for reliable evaluation of controlled protein release under comparable conditions.

Upon lowering the pH from 9.0 to 6.5, partial release of AGP from the magnetic nanocomposites was observed, confirming the reversible nature of boronic acid–diol interactions. Under the applied conditions, approximately 9–12% of the initially bound AGP was released within 30 min of incubation. These values are considered representative for mild, pH-triggered dissociation of boronate ester complexes in heterogeneous systems and are sufficient to demonstrate proof-of-concept reversibility.

It should be noted that in several literature reports, AGP or other glycoproteins are released from boronic acid-based materials under strongly acidic conditions (pH 4–5),^54^ which promote nearly complete dissociation of boronate ester bonds. However, such conditions may compromise protein stability and induce partial denaturation or irreversible conformational changes, particularly for sensitive glycoproteins such as AGP. In the present study, pH 6.5 was deliberately selected as a compromise between effective disruption of boronic acid–diol interactions and preservation of the structural integrity of the glycoprotein.

Overall, the observed partial release demonstrates that AGP binding to the magnetic nanocomposites is reversible and can be modulated by pH changes. Importantly, the applied release conditions prioritize protein stability over maximal release yield, which is critical for studies involving biologically relevant glycoproteins. The results therefore support the suitability of the magnetic nanocomposites as proof-of-concept platforms for reversible glycoprotein capture under mild conditions.

## 6 Perspective in the Future: Challenges and Constraints

From a practical perspective, the developed boronic acid-functionalized starch-based magnetic nanocomposites may serve as affinity materials for the enrichment or preconcentration of glycoproteins from simplified biological matrices. In comparison with widely reported cellulose- or dextran-based boronate affinity systems, the present materials introduce starch as a renewable, structurally distinct polysaccharide platform, offering greater conformational flexibility and tunable polymer architecture. These features allow modulation of the functional accessibility of boronic acid groups rather than relying solely on their total chemical content. At the same time, the scope of the present study should be clearly defined. The binding experiments were conducted under controlled conditions using single-protein systems, with the primary focus on elucidating structure–function relationships rather than on maximizing binding capacity or evaluating performance in complex protein mixtures. Accordingly, the results should be interpreted as a proof-of-concept illustrating how polymer architecture and functional accessibility govern glycoprotein binding behavior. Nevertheless, the ability to modulate glycoprotein binding and achieve partial release under mild pH conditions highlights the potential of starch-based boronic nanocomposites as model systems for studying reversible boronic acid–diol interactions and as a foundation for the future development of tailored affinity materials.

## 7 Conclusion

In summary, starch-based magnetic nanocomposites functionalized with boronic acids were developed and evaluated as systems capable of interacting with glycoproteins. Modified starch derivatives were successfully employed as coatings for magnetic nanoparticles, enabling the presentation of boronic acid groups on the material surface. To the best of our knowledge, starch-based magnetic nanomaterials functionalized with boronic acids have not been previously reported. The interaction of the obtained materials with α-1-acid glycoprotein was shown to depend on the accessibility of boronic acid groups and on environmental pH, consistent with the reversible nature of boronic acid–diol interactions. Among the investigated systems, CMS-PBA-MNPs exhibited higher glycoprotein binding capacities than DAS-PBA-MNPs, which was attributed to differences in polymer architecture and functional accessibility of boronic acid groups. Furthermore, only negligible binding of a nonglycosylated reference protein was observed under identical conditions, indicating that protein uptake was not dominated by nonspecific adsorption. Partial release of α-1-acid glycoprotein from the magnetic nanocomposites under mildly acidic conditions confirmed the reversibility of the binding interactions.

Overall, the presented results demonstrate that boronic acid-functionalized starch-based magnetic nanocomposites provide a tunable and well-defined proof-of-concept platform for studying pH-responsive and reversible interactions with glycoproteins under mild conditions, laying the groundwork for further investigations of boronate affinity systems.
